# Efficacy and safety of steroid injections for shoulder and elbow tendonitis: a meta-analysis of randomised controlled trials

**DOI:** 10.1136/ard.2008.099572

**Published:** 2008-12-03

**Authors:** C Gaujoux-Viala, M Dougados, L Gossec

**Affiliations:** 1 Paris Descartes University, Medicine Faculty; UPRES-EA 4058; APHP, Rheumatology B Department, Cochin Hospital, Paris France; 2 Université Montpellier I, EA 2415 Epidémiologie, Biostatistique, Santé Publique, Montpellier, France

## Abstract

**Objectives:**

To assess the efficacy and safety of steroid injections for patients with tendonitis of the shoulder or elbow.

**Methods:**

A systematic review of the literature using PubMed, EMBASE, the Cochrane library and manual searches was performed until April 2008. All randomised controlled trials (RCTs) reporting the efficacy on pain or functional disability, and/or the safety of steroid injections, versus placebo, non-steroidal anti-inflammatory drugs (NSAIDs) or physiotherapy in patients with tendonitis were selected. Pooled effect size (ES) was calculated by meta-analysis using the Mantel–Haenszel method.

**Results:**

In all, 20 RCTs were analysed (744 patients treated by injections and 987 patients treated by controls; 618 shoulders and 1113 elbows). The pooled analysis indicated only short-term effectiveness of steroids versus the pooled controls for pain and function (eg, pain at week 1–3 ES = 1.18 (95% CI 0.27 to 2.09), pain at week 4–8 ES = 1.30 (95% CI 0.55 to 2.04), pain at week 12–24 ES = −0.38 (95% CI −0.85 to 0.08) and pain at week 48 ES = 0.07 (95% CI −0.60 to 0.75)). Sensitivity analyses indicated similar results whatever the localisation, type of steroid and type of comparator except for NSAIDs: steroid injections were not significantly better than NSAIDs in the short-term. Steroid injections appeared more effective than pooled other treatments in acute or subacute tendonitis. The main side effects were transient pain after injection (10.7% of corticosteroid injections) and skin modification (4.0%).

**Conclusions:**

Steroid injections are well tolerated and more effective for tendonitis in the short-term than pooled other treatments, though similar to NSAIDs. No long-term benefit was shown.

Tendon lesions represent a large proportion of rheumatic issues resulting in pain and disability, and absence from work.^1^ The shoulder and elbow are the most frequent localisations.^2^ ^3^ The most common type of tendon lesion is tendinosis, which is an intratendinous degeneration and is the basis of epicondylitis, rotator cuff tendonitis or subacromial impingement.^4^ ^5^ The physiopathology of these disorders is different from adhesive capsulitis or full thickness rotator cuff tear.^4^ Tendon lesions are a therapeutic challenge for the general practitioner and the rheumatologist. There are a great variety of potential treatments, surgical intervention being the most radical. In usual practice, pain-relieving medications, non-steroidal anti-inflammatory drugs (NSAIDs), steroid injections and physical therapy are the most frequent options.^2^ Although local steroid injections are one of the most common treatments, there is no strong evidence to support their use^6^ and they have potential adverse effects.^7^ The optimum timing of steroid injections is also still unclear. Because of conflicting results, two meta-analyses were conducted several years ago, one in 1996^8^ and one in 2002 by the Cochrane group;^6^ however, this second analysis included various shoulder disorders (rotator cuff disease, adhesive capsulitis, full thickness rotator cuff tear and mixed diagnoses). Moreover the conclusion was not clear cut. In 2002, Smidt *et al*^9^ concluded that although the available evidence shows superior short-term effects of corticosteroid injections for lateral epicondylitis, it was not possible to draw firm conclusions on the effectiveness of injections, due to the lack of high quality studies. Therefore it appeared important to perform a new systematic review, including diagnostic considerations and recent trials.

The objective of the present study was to assess the efficacy on pain and functional disability, and to assess the safety of, steroid injections for patients with tendonitis of the shoulder and elbow in published randomised controlled trials (RCTs). The clinical question considered was whether or not steroid injections are effective compared with other commonly used treatments (“wait and see”, NSAIDs, physiotherapy) or placebo in terms of improvement of pain and functional ability in tendinosis (ie, epicondylitis, rotator cuff tendonitis or subacromial impingement).

## Methods

This meta-analysis was conducted according to the Cochrane Collaboration guidelines.^10^

### Study selection

A systematic literature search was performed in PubMed Medline, EMBASE and Cochrane library databases up to April 2008 without limitation of years of publication or journal, using the following keywords: ((“shoulder pain”[MeSH] OR “shoulder pain”[All Fields]) OR (“shoulder joint”[MeSH Terms] OR “shoulder joint”[All Fields]) OR (“rotator cuff”[MeSH Terms] OR “rotator cuff”[All Fields]) OR (“shoulder impingement syndrome”[MeSH Terms] OR “shoulder impingement syndrome”[All Fields] OR “subacromial impingement syndrome”[All Fields]) OR (epicondylitis [All Fields] OR “tennis elbow”[MeSH Terms] OR “tennis elbow”[All Fields]) OR (“tendon injuries”[MeSH Terms] OR “tendon injuries”[All Fields])) AND ((“steroids”[MeSH Terms] OR “steroids”[All Fields] OR “steroid”[All Fields]) OR (“glucocorticoids”[MeSH Terms] OR “glucocorticoids”[All Fields] OR “glucocorticoids”[Pharmacological Action])). The limits were English or French language and clinical trial or RCT. In addition, reference lists of the papers initially detected were manually searched to identify additional relevant reports.

The trials were initially selected on the basis of their titles and abstract, then on the full texts. The inclusion criteria were all RCTs reporting the efficacy on pain and/or function, and/or the safety of steroid injections versus placebo, “wait and see”, NSAIDs or physiotherapy in patients with epicondylitis, rotator cuff tendonitis or subacromial impingement. Articles reporting no interpretable results (data required included mean (SD)) for any of the three outcome measures (pain, function and safety) were not analysed.

### Data collection

One investigator (CGV) selected the articles and collected the data using a predetermined form. The following methodological features were collected: blinding, intention to treat analysis or not and number of participants who completed follow-up. The Jadad scale was applied,^11^ which contains two questions for randomisation and masking and one question evaluating the reporting of withdrawals and dropouts. Each question entails a yes or no response option. In total, 5 points can be awarded, with higher scores indicating higher quality. For each trial, demographic characteristics (sex, mean age), tendinosis features and duration, type of steroid (with doses and number of injections), type of comparator and duration of follow-up were collected. Acute/subacute and chronic disease were defined as having a symptom duration of <12 weeks versus ⩾12 weeks, respectively. Pain intensity was extracted from the studies, as available, by a 100 mm visual analogue scale (VAS), the Patient Related Forearm Evaluation Questionnaire (PREFQ)-pain,^12^ or other pain scores; physical function was extracted, as available, by the Disabilities of the Arm, Shoulder and Hand (DASH) score,^13^ the PREFQ-function,^12^ the total constant score,^14^ the limitation of function 10-point Likert scale,^15^ or the function scores (0–3 scale,^16^ 0–5 scale).^17^ Pain intensity and physical function assessed by different scores according to different studies were transformed linearly to fit the range 0–100, in which 0 was the best situation and 100 the worst. Efficacy was assessed by the change in overall pain intensity and/or physical function status between baseline and week 1 to 3, week 4 to 8, week 12 to 24 and week 48 (as available according to the studies) in steroid injection and control groups.

In order to evaluate safety, data were extracted from each study in active and control groups regarding the number and the type of all adverse events reported.

### Statistical analysis

In each trial the effect size (ES) or the standardised response mean (SRM) were determined to assess the magnitude of treatment effect. The ES is calculated as the ratio of the treatment effect (mean differences in treatment group minus differences in control group) to the pooled standard deviation of these differences.^18^ Improvement (eg, lower pain VAS) was considered as a positive change. This calculation entails the use of means for baseline and final data with a measure of variability such as SD. Every effort was made to calculate the ES in all studies. If the SD was given in only one group it was used as baseline SD for both groups. However, if no measure of variability was given the ES could not be extrapolated and we calculated the SRM (mean change divided by SD of the change) when available. By convention, an ES <0.2 is usually considered as trivial; 0.2–0.5 as small; 0.5–0.8 as moderate; 0.8–1.2 as important and >1.2 as very important.^19^ SRM values >0.8 are considered as large. Pooled ES and pooled SRM were calculated by meta-analysis, using the Mantel–Haenszel method. The RevMan V.4.2 statistical software (Review Manager, Copenhagen, Denmark) was used. Primary analyses examined pooled ES and pooled SRM of steroid injections versus controls for pain intensity and physical function. Sensitivity analyses were calculated within subgroups of studies decided a priori (joint involved, duration of pain, type of steroid and type of comparator) to assess the robustness of the main conclusions. Statistical heterogeneity was tested by Q test (χ^2^).^20^ All meta-analyses were carried out with use of random effects model in case of significant heterogeneity. The number needed to harm (NNH) was used to assess steroid injections safety. Harm was primarily defined by the occurrence of one or more adverse events. The NNH is the number of patients that should be treated to observe the occurrence of one extra adverse event in the treatment group compared to the control group. The advantage of the NNH is that it reflects an absolute risk increase, and because it is related to the control event rate, it reflects the true baseline or underlying risk of the study population.^21^ For rational decision making in daily clinical practice, absolute measures such as NNH may be more meaningful than relative measures.^22^ Because of the large and non-significant confidence intervals around the steroid injection adverse event rates, confidence intervals were not reported for the NNH, as proposed by McQuay and Moore.^23^

## Results

### Literature search results and trials characteristics

Initially, 218 potentially relevant articles were screened. From them, 199 were excluded. Finally, after manually searching the reference lists, 20 randomised trials were included: 16 for efficacy and 19 for safety (selection process shown in Supplementary material). Thus, this systematic review included 1731 patients; 744 (43.0%) patients treated by injection and 987 (57.0%) patients treated by other treatments (n = 570) or placebo (n = 417). A total of 618 (35.7%) presented with a shoulder lesion and 1113 (64.3%) with an elbow lesion. The mean (SD) age of these patients was 47.6 (4.8) years and 694 (38%) were women. Patient characteristics are detailed in table 1 and trial characteristics in the Supplementary material.

**Table 1:** Characteristics of patients with tendonitis included in a meta-analysis of randomised controlled trials of steroid injections for shoulder and elbow tendonitis

	All patients	Injection group (steroids)	Control group
Number of patients	1731	744	987
Mean (SD) age, years	47.5 (4.9)	48.2 (5.2)	47.3 (5.8)
Female sex, n (%)	659 (38)	NA	NA
Number of patients with shoulder lesion: rotator cuff tendonitis and subacromial impingement (%)	618 (33.9)	315	303
Acute	−253 (40.9)	−122	−131
Chronic	−245 (39.6)	−103	−142
Heterogeneous	−120 (19.4)	−90	− 30
Number of patients with elbow lesion: epicondylitis (%)	1113 (64.3)	429	684
Acute	−513 (46.1)	−168	−345
Chronic	−483 (43.4)	−208	−275
Heterogeneous	−117 (10.5)	−53	−64

Acute: <12-week symptom duration; chronic: ⩾12-week symptom duration, heterogeneous: variable symptom duration.

NA, not available.

The methodological quality was correct: the mean (SD) Jadad score was 3.1 (0.9) (range 1–5). In all, 13 (65%) trials performed intention to treat (ITT) analyses (see Supplementary material). Of the 16 trials for efficacy, 9 included a flowchart or described precisely the patient selection process and outcome, 14 used a concealed random allocation and 11 presented differences in change with confidence intervals, 2 with interquartile range and 2 with standard error of the mean.

### Efficacy of steroid injections

Of the 20 RCTs analysed, 16 provided the required data to calculate the efficacy on pain intensity for 511 participants who received steroid injections and for 763 who received a comparator. Eight studies concerned shoulder tendinosis and eight epicondylitis. The data on function were extracted from 7 RCTs: 4 concerning the shoulder and 3 concerning the elbow (a total of 614 patients). The characteristics of these trials are summarised in the Supplementary material and the ES or SRM for pain intensity and physical function at different end points (week 1 to 3, week 4 to 8, week 12 to 24 and week 48) are detailed in table 2.

**Table 2:** Effect size and standardised response mean of controlled trials comparing steroid injections with placebo or other comparator for pain intensity and physical function in patients with tendonitis

Reference	Comparator	Outcome measure	Week 1–3	Week 4–8	Week 12–24	Week 48
Vecchio *et al* (1993)^24^	Placebo	Pain VAS	**SRM = 1.16 (0.58 to 1.72)**	**SRM = 0.90 (0.34 to 1.44)**	SRM = 0.00 (−0.53 to 0.53)	NA
Adebajo *et al* (1990)^16^	Placebo	Pain VAS	NA	**SRM = 1.09 (0.40 to 1.73)**	NA	NA
		Limitation of function (0–3 scale)	NA	**SRM = 3.22 (2.23 to 4.08)**	NA	NA
	Oral diclofenac	Pain VAS	NA	SRM = 0.43 (−0.21 to 1.05)	NA	NA
		Limitation of function (0–3 scale)	NA	SRM = 0.00 (−0.62 to 0.62)	NA	NA
White *et al* (1986)^25^	Indomethacine	Pain VAS	NA	ES = −0.21 (−0.83 to 0.41)	NA	NA
Berry *et al* (1980)^26^	Placebo	Pain VAS	ES = −0.17 (−0.96 to 0.64)	ES = −0.68 (−1.48 to 0.16)	NA	NA
	Physiotherapy	Pain VAS	ES = 0.14 (−0.67 to 0.93)	ES = 0.19 (−0.62 to 0.99)	NA	NA
Petri *et al* (1987)^17^	Placebo	Pain score	SRM = 0.45 (−0.12 to 1.00)	SRM = 0.66 (0.08 to 1.22)	NA	NA
		Limitation of function (0–5 scale)	**SRM = 0.64 (0.06 to 1.19)**	SRM = 0.39 (−0.17 to 0.95)	NA	NA
	Naproxen	Pain score	SRM = 0.11 (−0.45 to 0.66)	SRM = 0.12 (−0.43 to 0.68)	NA	NA
		Limitation of function (0–5 scale)	SRM = 0.10 (−0.46 to 0.65)	SRM = −0.05 (−0.60 to 0.51)	NA	NA
Alvarez *et al* (2005)^13^	Placebo	Pain VAS	ES = 0.26 (−0.24 to 0.76)	ES = −0.29 (−0.78 to 0.22)	ES = −0.41 (−0.93 to 0.11)	NA
		DASH	ES = 0.19 (−0.31 to 0.69)	ES = −0.09 (−0.58 to 0.41)	ES = −0.27 (−0.76 to 0.24)	NA
McInerney *et al* (2003)^27^	Wait and see	Pain VAS	NA	NA	ES = 0.10 (−0.31 to 0.52)	NA
Akgün *et al* (2004)^14^	Placebo	Pain VAS	NA	ES = 0.71 (−0.02 to 1.40)	ES = 0.28 (−0.42 to 0.97)	NA
		Total Constant score	NA	ES = 0.35 (−0.36 to 1.04)	ES = 0.09 (−0.61 to 0.78)	NA
Lewis *et al* (2005)^28^	Placebo	Pain VAS	**SRM = 7.08 (6.04 to 8.03)**	NA	NA	NA
	Naproxen	Pain VAS	**SRM = 5.43 (4.57 to 6.21)**	NA	NA	NA
Hay *et al* (1999)^15^	Placebo	Pain VAS	NA	**ES = 1.57 (1.14 to 1.99)**	ES = 0.00 (−0.37 to 0.37)	ES = 0.00 (−0.37 to 0.37)
		Impairment of function (10 point Likert scale)	NA	**ES = 0.90 (0.50 to 1.28)**	ES = −0.22 (−0.6 to 0.15)	ES = 0.00 (−0.37 to 0.37)
	Naproxen	Pain VAS	NA	**ES = 2.07 (1.58 to 2.52)**	**ES = 0.41 (0.03 to 0.80)**	**ES = 0.41 (0.03 to 0.80)**
		Impairment of function (10 point Likert scale)	NA	**ES = 1.35 (0.92 to 1.76)**	**ES = −0.45 (−0.83 to −0.06)**	ES = 0.00 (−0.38 to 0.38)
Smidt *et al* (2002)^29^	Wait and see	Pain VAS	**SRM = 1.51 (1.09 to 1.90)**	**SRM = 1.49 (1.08 to 1.88)**	SRM = 0.3 (−0.06 to 0.66)	SRM = −0.15 (−0.51 to 0.20)
		Elbow disability	**SRM = 1.45 (1.04 to 1.84)**	**SRM = 1.46 (1.05 to 1.85)**	SRM = 0.10 (−0.26 to 0.45)	SRM = −0.36 (−0.72 to 0.00)
	Physiotherapy	Pain VAS	**SRM = 1.49 (1.08 to 1.87)**	**SRM = 1.21 (0.82 to 1.58)**	SRM = −0.04 (−0.39 to 0.31)	**SRM = −0.41 (−0.76 to −0.05)**
		Elbow disability	**SRM = 1.21 (0.82 to 1.58)**	**SRM = 1.13 (0.74 to 1.50)**	SRM = −0.23 (−0.58 to 0.12)	**SRM = −0.58 (−0.93 to −0.22)**
Price *et al* (1991)^30^	Placebo	Pain VAS	**NA**	**ES = 6.37 (4.98 to 7.58)**	**ES = −2.21 (−2.85 to −1.50)**	NA
Bisset *et al* (2006)^31^	Wait and see	Pain VAS	**ES = 1.52 (1.12 to 1.90)**	**ES = 1.18 (0.80 to 1.54)**	**ES = −0.50 (−0.84 to −0.15)**	**ES = −0.64 (−0.99 to −0.29)**
	Physiotherapy	Pain VAS	**ES = 0.99 (0.63 to 1.35)**	**ES = 0.56 (0.21 to 0.90)**	**ES = −0.81 (−1.16 to −0.45)**	**ES = −0.76 (−1.11 to −0.40)**
Tonks *et al* (2006)^12^	Wait and see	PREFQ-pain	NA	**SRM = 1.96 (0.88 to 2.89)**	NA	NA
		PREFQ-function	NA	**SRM = 1.31 (0.34 to 2.17)**	NA	NA
	Physiotherapy	PREFQ-pain	NA	**SRM = 1.20 (0.18 to 2.11)**	NA	NA
		PREFQ-function	NA	**SRM = 1.45 (0.40 to 2.38)**	NA	NA
Saartok *et al* (1986)^32^	Naproxen	Pain score (0–8)	ES = −0.05 (−0.90 to 0.81)	**NA**	NA	NA
Stahl *et al* (1997)^33^	Placebo	Pain VAS	NA	**ES = 3.60 (2.74 to 4.36)**	NA	**ES = 1.2 (0.63 to 1.73)**

Results are presented as effect (95% CI). Significant results are in bold type.

DASH, Disabilities of the Arm, Shoulder and Hand; ES, effect size; NA, not available; PREFQ, Patient Related Forearm Evaluation Questionnaire; SRM, standardised response mean; VAS, visual analogue scale.

**Figure 1:**
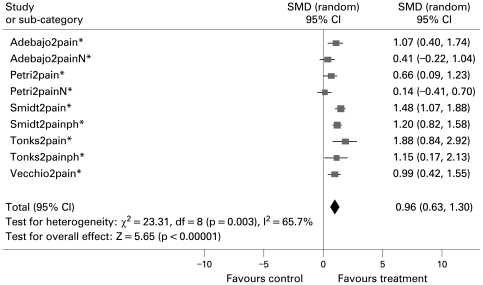
Pain standardised response mean (SRM) at weeks 4–8 versus all comparators.

**Figure 2:**
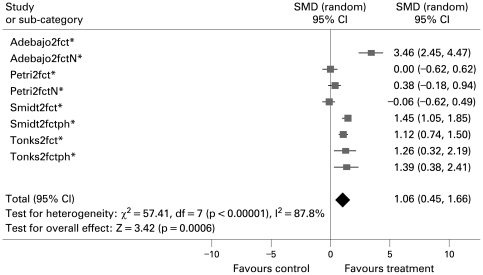
Function standardised response mean (SRM) at weeks 4–8 versus all comparators.

The pooled analysis is shown table 3, and indicated short-term effectiveness (1–3 weeks and 4–8 weeks) of steroids (figs 1 and 2). At longer-term follow-up no difference for pain could be detected, and steroid injections appeared less effective particularly on functional disability than pooled other treatments.

**Table 3:** Pooled effect size (ES) and pooled standardised response mean (SRM) for steroid injections in tendonitis according to the comparator

Comparator	Outcome	Week 1–3	Week 4–8	Week 12–24	Week 48
All	Pain ES	**1.18 (0.27 to 2.09)**	**1.30 (0.55 to 2.04)**	−0.38 (−0.85 to 0.08)	0.07 (−0.60 to 0.75)
	Pain SRM	**2.45 (1.10 to 3.80)**	**0.96 (0.63 to 1.30)**	0.10 (−0.12 to 0.33)	**−0.28 (−0.53 to −0.03)**
	Function ES	0.20 (−0.30 to 0.70)	**0.66 (0.03 to 1.30)**	**−0.27 (−0.49 to −0.04)**	0.00 (−0.27 to 0.27)
	Function SRM	**0.88 (0.32 to 1.44)**	**1.06 (0.45 to 1.66)**	−0.07 (−0.39 to 0.25)	**−0.47 (−0.72 to −0.22)**
Placebo	Pain ES	**1.89 (0.20 to 3.59)**	**1.67 (0.56 to 2.78)**	−0.45 (−1.01 to 0.11)	0.25 (−0.82 to 1.32)
	Pain SRM	**2.52 (0.58 to 4.45)**	**1.16 (0.79 to 1.53)**	0.21 (−0.09 to 0.50)	−0.15 (−0.51 to 0.20)*
	Function ES	0.20 (−0.30 to 0.70)*	0.41 (−0.24 to 1.07)	−0.17 (−0.45 to 0.11)	0.00 (−0.37 to 0.37)*
	Function SRM	**1.08 (0.30 to 1.85)**	**1.57 (0.55 to 2.59)**	0.09 (−0.26 to 0.45)*	−0.36 (−0.72 to 0.00)*
Physiotherapy	Pain ES	0.64 (−0.18 to 1.47)	**0.51 (0.19 to 0.84)**	−0.83 (−1.18 to −0.47)*	−0.74 (−1.10 to −0.39)*
	Pain SRM	1.48 (1.08 to 1.87)*	**1.19 (0.84 to 1.55)**	−0.04 (−0.39 to 0.31)*	−0.40 (−0.76,−0.05)*
	Function ES	NE	NE	NE	NE
	Function SRM	1.20 (0.82 to 1.58)*	**1.15 (0.80 to 1.51)**	−0.23 (−0.58 to 0.12)*	−0.57 (−0.93 to −0.22)*
NSAID	Pain ES	−0.04 (−0.90 to 0.82)*	0.95 (−1.31 to 3.21)	0.42 (0.03 to 0.80)*	0.42 (0.03 to 0.80)*
	Pain SRM	2.88 (−2.64 to 8.40)	0.26 (−0.16 to 0.68)	NE	NE
	Function ES	NE	1.37 (0.94 to 1.79)*	−0.46 (−0.84 to −0.07)*	0.00 (−0.38 to 0.38)*
	Function SRM	0.10 (−0.45 to 0.66)*	−0.04 (−0.45 to 0.38)	NE	NE

Results are presented as effect (95% CI). Significant results are in bold type.

*One study.

NE, not estimable; NSAID, non-steroidal anti-inflammatory drug.

### Heterogeneity

The heterogeneity was substantial for pain, and to a lesser extent for function, at week 1–3 and at week 4–8 (for example pain ES: Q = 99.25 for 6 degrees of freedom; p<0.001 and Q = 197.35 for 10 degrees of freedom; p<0.001). The other results were homogeneous.

### Sensitivity analyses: location and steroid used

In the assessment of heterogeneity, sensitivity analyses showed no changes in the conclusions according to the localisation, or the type of steroid (data not shown).

### Sensitivity analyses: type of comparator

Steroid injections were more efficient in the short-term (week 1 to 3 and week 4 to 8) than different comparators (table 3). However the pooled ES versus NSAIDs were not significant in the short-term; long-term results were significant but are issued from a single study.^16^ Moreover steroid injection was shown to have a more important benefit on pain compared to placebo than to physiotherapy: pain at week 4–8 versus placebo ES = 1.67 (95% confidence interval (CI) 0.56 to 2.78) versus pain at week 4–8 versus physiotherapy ES = 0.51 (95% CI 0.19 to 0.84) (p<0.001).

### Sensitivity analyses: disease duration

This meta-analysis suggested that steroid injections are more effective in acute or subacute tendonitis (duration of symptoms below 12 weeks) than in chronic disease: pain at week 1–3 SRM = 3.35 (95% CI 1.63 to 5.08) versus 0.37 (95% CI −0.17 to 0.92) (p<0.001).

### Safety of steroid injections

In all, 19 studies (no. of patients = 1754) provided suitable data to assess corticosteroid injection safety. The main side effects were transient pain after injection (10.7% of corticosteroid injection) and skin atrophy or depigmentation (4.0%). There were no treatment discontinuations for toxicity. The NNH for steroid injection versus other commonly used treatments was 26 (ie, 26 patients would need to be treated to observe the occurrence of 1 extra adverse event in the injection group) compared to all the comparators, and versus placebo it was 9; however, the confidence intervals were not significant. The NNH values versus each comparator and calculated for each adverse event are provided in table 4.

**Table 4:** Adverse events (AE) reported for steroid injections versus placebo or other comparator

Adverse event	No (%) of AE in patients treated with corticoids*	Comparator	No (%) of AE patients treated with comparator*	NNH
All	121/713 (16.97)	Placebo	34/566 (6.01)	9
		Physiotherapy	80/203 (39.41)	−4
		NSAID	11/181 (6.08)	9
Pain	76/713 (10.66)	Placebo	19/566 (3.36)	14
		Physiotherapy	54/203 (26.60)	−6
		NSAID	0/181 (0)	9
Skin atrophy and depigmentation	29/713 (4.07)	Placebo	5/566 (0.88)	31
		Physiotherapy	6/203 (2.96)	90
		NSAID	0/181 (0)	25
Gastrointestinal upset	1/713 (0.14)	Placebo	1/566 (0.18)	−2500
		Physiotherapy	0/203 (0)	714
		NSAID	7/181 (3.87)	−27
Facial flush	3/713 (4.21)	Placebo	0/566 (0)	24
		Physiotherapy	0/203 (0)	24
		NSAID	0/181 (0)	24

*In studies where the AE was reported.

NNH, number needed to harm; NSAID, non-steroidal anti-inflammatory drug.

## Discussion

This meta-analysis indicated short-term effectiveness (1–3 weeks and 4–8 weeks) of steroid injections in shoulder and elbow tendinosis on pain and functional disability, but at longer-term follow-up no difference for pain could be detected and steroid injections appeared less effective for function than pooled other treatments. Compared to NSAIDs however, steroid injections did not appear statistically more efficacious in the short term.

There is little consensus on the optimum timing of corticosteroid injections in tendonitis, relative to the symptom duration. Some experts advocate injections when the patient does not respond to a certain period of rest (2 or 3 months),^34^ whereas others argue that injectable steroids should be deferred as long as possible.^35^ However, this meta-analysis has shown that steroid injections are more effective in acute or subacute tendonitis (duration of symptoms below 12 weeks) than in chronic disease. Therefore our results indicate that the optimum timing for steroid injections may be in the early weeks of tendonitis symptoms.

Despite the high level of interest in the aetiologies, diagnosis and treatment of tendonitis in the literature, there are relatively few randomised controlled trials and the outcomes used are heterogeneous. Physical function was rarely assessed by the same score in different studies. In some studies the ES or the SRM could not be extrapolated either due to lack of data or to inappropriate outcomes (eg, qualitative evaluation). Another limitation of this review is possible publication bias (ie, negative trials are often unpublished, which may have overestimated the beneficial effect of corticosteroid injections).

Several reviews on the utility of steroid injections in shoulder pain have been performed and conflicting results found, but the studies analysed assessed a wide variety of conditions and disorders (including frozen shoulder) and there was often no pooling of results.^36^ ^37^ A Cochrane review found that subacromial steroid injection was effective in improving the range of abduction. Arrol *et al*^38^ conducted a meta-analysis using a binary outcome (“improvement or not”) and concluded that subacromial injections of steroids are effective for improvement for rotator cuff tendonitis up to a 9-month period, and that they are also probably more effective than NSAIDs. Concerning epicondylitis, Labelle *et al*^39^ concluded that there was insufficient scientific evidence to support the use of corticosteroid injections. In 1996, Assendelft *et al*^8^ conducted a meta-analysis using a binary outcome (“success or failure”). The pooled analysis indicated short-term effectiveness of steroid injections.

However, this review differs from these earlier works in several important respects. Firstly, it included diagnostic considerations and attempted to differentiate studies based upon the nature of the populations being studied, recognising that the benefits of treatment may vary for different underlying causes of shoulder or elbow pain. This analysis was thus centred on tendinosis. Secondly, steroid injections were compared to placebo but also to other commonly used treatments (“wait and see”, NSAIDs, physiotherapy) and this heterogeneity in comparators was taken into account, leading to sensitivity analyses. Thirdly, effect sizes were calculated for the different reported outcome measures in different trials. This enabled a direct comparison between studies using the same scale, whatever the outcome measure. If the ES could not be calculated, another pooled measure of effect, the SRM, was calculated when available in order not to lose information. Fourthly, time since injections was taken into account, as efficacy was assessed by the change in overall pain intensity and/or physical functional status between baseline and week 1 to 3, week 4 to 8, week 12 to 24 and week 48. In spite of these measures, statistical testing revealed heterogeneity during the first weeks. The a priori defined sensitivity analyses showed no changes in the main conclusions with the localisation, duration of pain, or type of corticoid. Some other possible reasons for this heterogeneity are variation in study quality (but only three Jadad scores were lower than 3), differences in the type of patients included or the composition of the various injection fluids, variation in the number and interval of injections (four studies allowed up to two injections and two studies up to three injections) and different methods of outcome measurement. In addition, diagnostic accuracy is always a difficulty in particular for shoulder disorders: selection may have influenced the results, as patients who were heterogeneous may have been included in the original RCTs.

Accuracy of placement of the needle for injections may also be an issue. Eustace *et al* indicated that, even in the hands of musculoskeletal specialists, only a minority of injections for shoulder pain are performed accurately (29% of subacromial injections) and that outcome significantly correlates with accuracy of injection.^40^ In the present meta-analysis, radiographic confirmation of appropriate needle placement and adequate technique was carried out only on the first 10 patients in the study by Alvarez *et al*.^13^

Although steroid injections were more efficacious in the first weeks than pooled comparators, importantly, steroid injections did not demonstrate superiority in the first weeks either for pain or for functional disability compared to NSAIDs. This is an important finding since NSAIDs are a widely used and relatively safe therapeutic option in tendonitis, leading to questions regarding the role of steroid injections in these pathologies. In usual care, steroid injections are in fact most often performed after NSAIDs have been prescribed but without sufficient efficacy. It should be noted that none of the studies included in this meta-analysis did in fact test this treatment strategy.

Adverse clinical effects of steroid injections were systematically investigated in this meta-analysis. This study indicates steroid injections are well tolerated, with infrequent and minor side effects. The main side effect was transient pain after injection (10.7% of corticosteroid injections). Haker *et al* assumed that postinjection pain is caused by the volume effect of the injection and the corticosteroid itself.^41^ Price *et al* reported a much lower percentage of postinjection pain for local anaesthetics (11%) than for corticosteroids (50%) for the same volume, indicating a possibly specific irritation caused by steroids.^30^ Skin atrophy and depigmentation were the second most frequently reported side effects (4%). Although most side effects are rare and temporary, skin atrophy and depigmentation can be permanent and should be explained to the patient before performing an injection. Although there are clinical reports of tendon ruptures following steroid injections,^42^ ^43^ no study reported this adverse event. There is strong evidence to conclude that the risks of serious adverse effects such as tendon rupture and infections after steroid injections for the indications and populations included and with the doses given, are very small.

In summary, these findings suggest that injections of corticosteroids are well tolerated and effective compared with other commonly used treatments (wait and see, physiotherapy) or placebo in terms of improvement of pain and function in tendinosis (epicondylitis, rotator cuff tendonitis or subacromial impingement) in the short term, however no long-term benefit was shown by this meta-analysis. Moreover there appears to be no benefit of steroid injections compared to NSAIDs. This study may lead the doctor to systematically prescribe a course of NSAIDs (if there are no contraindications) before potentially performing a steroid injection. Moreover it may lead the doctor to perform steroid injections mostly in acute or subacute tendonitis, where the potential benefit appears clearer than in chronic disease.
